# Supplementary material for: Atg8 orchestrates stress-responsive chromatin programs across immunity and metabolism
Source: bioRxiv. 2026 May 27:2026.05.22.727304. Preprint. [Version 2] doi: 10.64898/2026.05.22.727304 (PMC13232312; doi:10.64898/2026.05.22.727304)
Supplement: Supplement 1 [file NIHPP2026.05.22.727304v2-supplement-1.pdf]

bioRxiv preprint doi: https://doi.org/10.64898/2026.05.22.727304; this version posted May 28, 2026. The copyright holder for this preprint (which was not certified by peer review) is the author/funder, who has granted bioRxiv a license to display the preprint in perpetuity. It is made available under a CC-BY 4.0 International license.

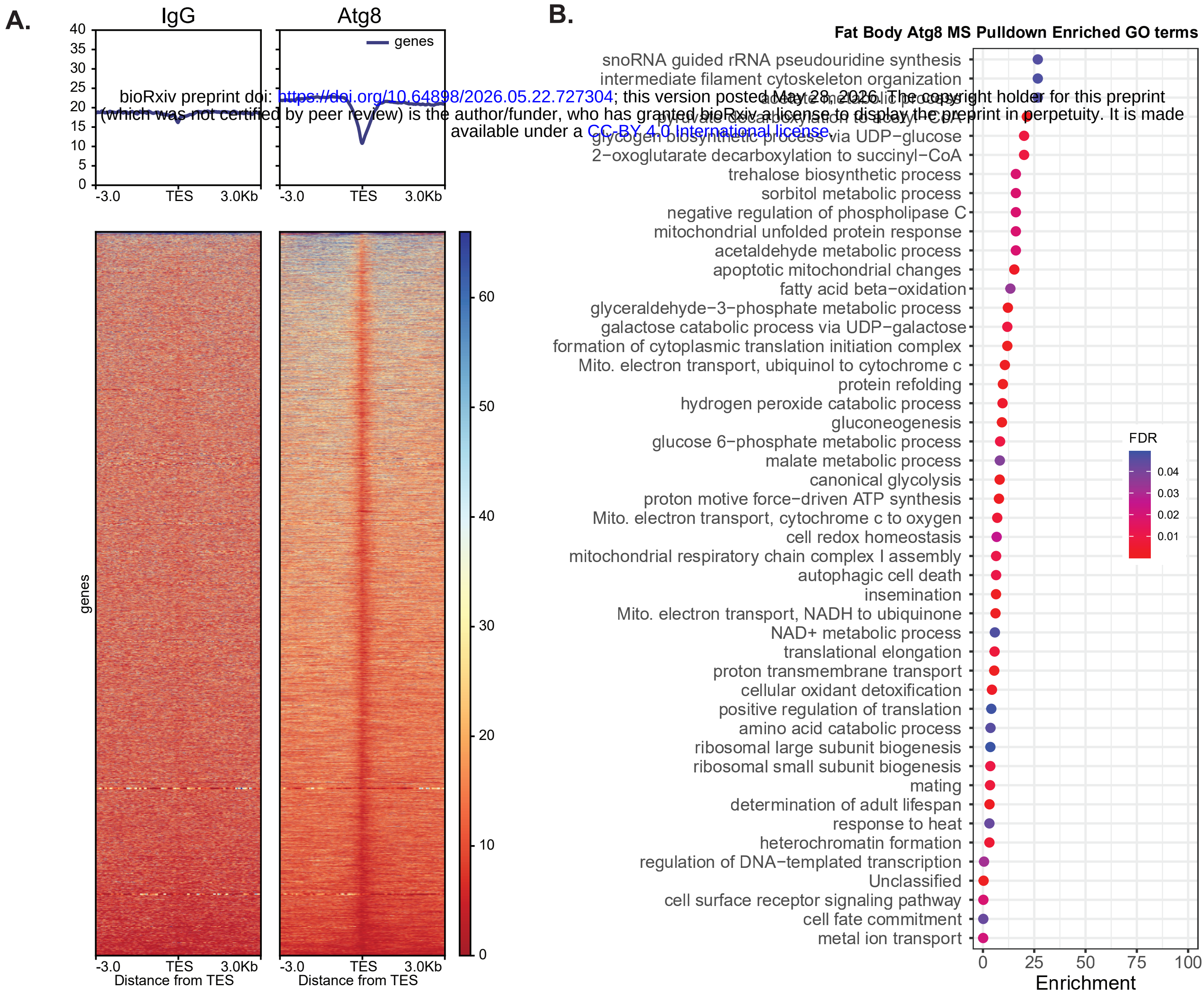

bioRxiv preprint doi: https://doi.org/10.64898/2026.05.22.727304; this version posted May 28, 2026. The copyright holder for this preprint (which was not certified by peer review) is the author/funder, who has granted bioRxiv a license to display the preprint in perpetuity. It is made available under a CC-BY 4.0 International license.

Figure 1 Supplement. Atg8 TES occupancy and protein association. A) overview of Atg8 chromatin binding in *W1118* 7-day-old adult flies on ND aligned to 5' transcription end sites (TES), compared to IgG negative control. The heatmap depicts the number of sequences relative to other TES in arbitrary units, with each row being a gene. Top graphs plot the mean occupancy across all TES within +/- 3kb. B) GO enrichment analysis of pathways of proteins pulled down with Atg8 from *Drosophila* fat bodies.

bioRxiv preprint doi: https://doi.org/10.64898/2026.05.22.727304; this version posted May 28, 2026. The copyright holder for this preprint (which was not certified by peer review) is the author/funder, who has granted bioRxiv a license to display the preprint in perpetuity. It is made available under a CC-BY 4.0 International license.

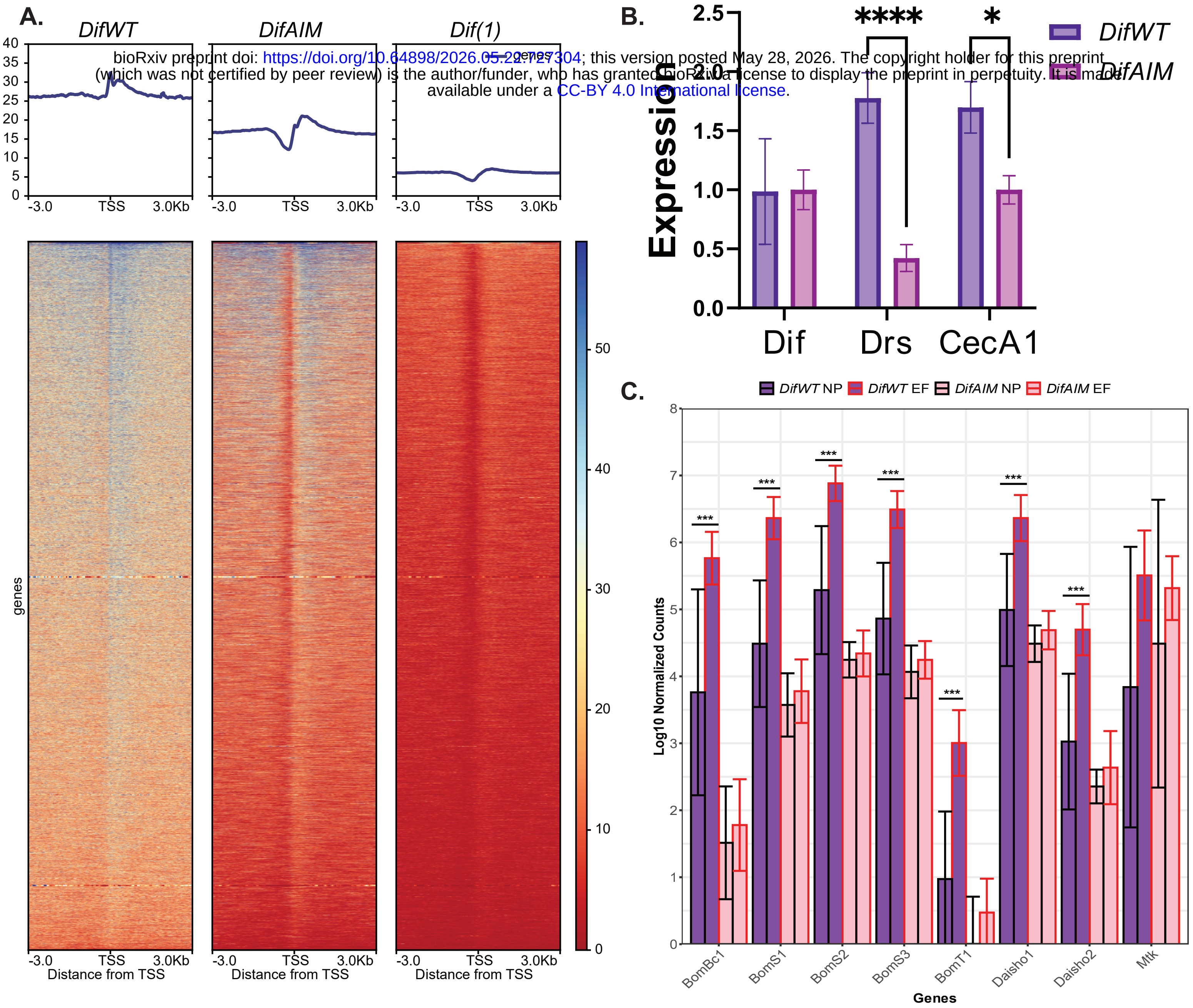

bioRxiv preprint doi: https://doi.org/10.64898/2026.05.22.727304; this version posted May 28, 2026. The copyright holder for this preprint (which was not certified by peer review) is the author/funder, who has granted bioRxiv a license to display the preprint in perpetuity. It is made available under a CC-BY 4.0 International license.

Figure 3 Supplement. A) Full heatmap of Dif Occupancy at TSS in *DifWT*, *DifAIM*, and *Dif(1)* flies. B) qPCR analysis showing reduced basal expression of Dif target AMPs (*Drosomycin* and *Cecropin A1*) in *DifAIM* (pink) compared to *DifWT* (purple) flies. C) full list of Gram-positive AMPs enriched in RNAseq Differential Expression data in *DifWT* infected vs uninfected which are unresponsive in *DifAIM* infected vs uninfected (padj<0.05). Error bars indicate standard deviation. Significance was determined by two-way ANOVA with Bonferroni post hoc correction. *p<0.5,**p<0.1,***p<0.01,****p<0.001.

bioRxiv preprint doi: https://doi.org/10.64898/2026.05.22.727304; this version posted May 28, 2026. The copyright holder for this preprint (which was not certified by peer review) is the author/funder, who has granted bioRxiv a license to display the preprint in perpetuity. It is made available under a CC-BY 4.0 International license.

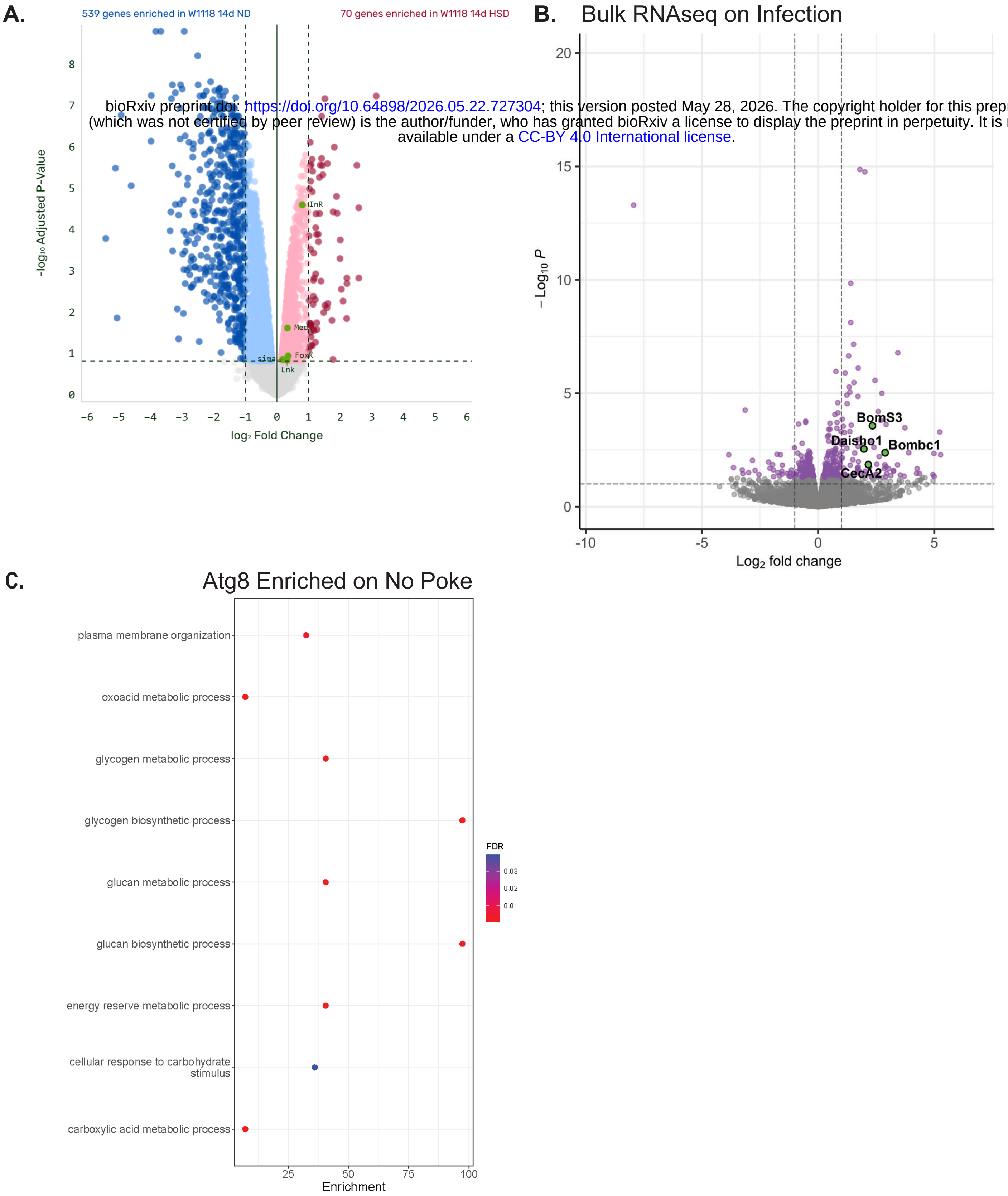

bioRxiv preprint doi: https://doi.org/10.64898/2026.05.22.727304; this version posted May 28, 2026. The copyright holder for this preprint (which was not certified by peer review) is the author/funder, who has granted bioRxiv a license to display the preprint in perpetuity. It is made available under a CC-BY 4.0 International license.

Figure 4 Supplement. A) Volcano plot of RNAseq data of *W1118* flies in 14d ND vs HSD and B) *DifWT* flies no poke vs *E. Faecalis* 6 hours post infection. C) GO analysis of genes found to be significantly upregulated and bound by Atg8 in No Poke flies compared to *E. faecalis*-infected flies.
